# Provider Interaction With an Electronic Health Record Notification to Identify Eligible Patients for a Cluster Randomized Trial of Advance Care Planning in Primary Care: Secondary Analysis

**DOI:** 10.2196/41884

**Published:** 2023-05-12

**Authors:** Jessica E Ma, Jared Lowe, Callie Berkowitz, Azalea Kim, Ira Togo, R Clayton Musser, Jonathan Fischer, Kevin Shah, Salam Ibrahim, Hayden B Bosworth, Annette M Totten, Rowena Dolor

**Affiliations:** 1 Geriatric Research, Education, and Clinical Center Durham Veterans Affairs Health Care System Durham, NC United States; 2 Division of General Internal Medicine Department of Medicine Duke University School of Medicine Durham, NC United States; 3 Division of General Medicine & Clinical Epidemiology Department of Medicine University of North Carolina School of Medicine Chapel Hill, NC United States; 4 Division of Hematology and Oncology Department of Medicine University of North Carolina School of Medicine Chapel Hill, NC United States; 5 Duke Office of Clinical Research Durham, NC United States; 6 Department of Community & Family Medicine Duke University School of Medicine Durham, NC United States; 7 Duke Population Health Management Office Durham, NC United States; 8 Department of Medicine Duke University School of Medicine Durham, NC United States; 9 Duke Health Performance Services Duke University Health System Durham, NC United States; 10 Department of Psychiatry and Behavioral Services Duke University School of Medicine Durham, NC United States; 11 Department of Population Health Sciences Duke University School of Medicine Durham, NC United States; 12 Center of Innovation to Accelerate Discovery and Practice Transformation Durham Veterans Affairs Health Care System Durham, NC United States; 13 Oregon Rural Practice Based Research Network Oregon Health & Science University School of Medicine Portland, OR United States

**Keywords:** advance care planning, electronic health record, notification, EHR, provider interaction, primary care, clinical study, referral, notifications, alerts

## Abstract

**Background:**

Advance care planning (ACP) improves patient-provider communication and aligns care to patient values, preferences, and goals. Within a multisite Meta-network Learning and Research Center ACP study, one health system deployed an electronic health record (EHR) notification and algorithm to alert providers about patients potentially appropriate for ACP and the clinical study.

**Objective:**

The aim of the study is to describe the implementation and usage of an EHR notification for referring patients to an ACP study, evaluate the association of notifications with study referrals and engagement in ACP, and assess provider interactions with and perspectives on the notifications.

**Methods:**

A secondary analysis assessed provider usage and their response to the notification (eg, acknowledge, dismiss, or engage patient in ACP conversation and refer patient to the clinical study). We evaluated all patients identified by the EHR algorithm during the Meta-network Learning and Research Center ACP study. Descriptive statistics compared patients referred to the study to those who were not referred to the study. Health care utilization, hospice referrals, and mortality as well as documentation and billing for ACP and related legal documents are reported. We evaluated associations between notifications with provider actions (ie, referral to study, ACP not documentation, and ACP billing). Provider free-text comments in the notifications were summarized qualitatively. Providers were surveyed on their satisfaction with the notification.

**Results:**

Among the 2877 patients identified by the EHR algorithm over 20 months, 17,047 unique notifications were presented to 45 providers in 6 clinics, who then referred 290 (10%) patients. Providers had a median of 269 (IQR 65-552) total notifications, and patients had a median of 4 (IQR 2-8). Patients with more (over 5) notifications were less likely to be referred to the study than those with fewer notifications (57/1092, 5.2% vs 233/1785, 13.1%; *P*<.001). The most common free-text comment on the notification was lack of time. Providers who referred patients to the study were more likely to document ACP and submit ACP billing codes (*P*<.001). In the survey, 11 providers would recommend the notification (n=7, 64%); however, the notification impacted clinical workflow (n=9, 82%) and was difficult to navigate (n=6, 55%).

**Conclusions:**

An EHR notification can be implemented to remind providers to both perform ACP conversations and refer patients to a clinical study. There were diminishing returns after the fifth EHR notification where additional notifications did not lead to more trial referrals, ACP documentation, or ACP billing. Creation and optimization of EHR notifications for study referrals and ACP should consider the provider user, their workflow, and alert fatigue to improve implementation and adoption.

**Trial Registration:**

ClinicalTrials.gov NCT03577002; https://clinicaltrials.gov/ct2/show/NCT03577002

## Introduction

Health care providers and health systems are increasingly recognizing the importance of advance care planning (ACP) [1]. ACP is the process of understanding a patient’s preferences around future medical care and how health care can best align with their personal values and goals [2]. Despite benefits of ACP, including improved quality of life, positive family outcomes, and goal-consistent care, the overall documentation of ACP remains low [3-5].

There are significant barriers to engaging patients in ACP across all practice settings, such as time limitations, provider skill set, and patient selection [6-9]. In primary care, one challenge is identifying the patients most likely to benefit from ACP, so that limited resources and time may be prioritized. Related to this challenge is determining how to best notify providers which patients have been identified for ACP interventions. One approach is using a notification via the electronic health record (EHR) to prompt providers to consider having an ACP conversation [10,11].

The approach of “prescreening” patients using the EHR and alerting providers to patient study eligibility is frequently used in clinical trials and offers an efficient and effective alternative to manually screening patient records [12-14]. We used a single EHR notification tool as both a clinical trial alert for participant recruitment and a prompt for providers to engage in ACP]. Although EHR notifications can add value to clinical workflows, EHR and associated notifications can contribute to provider fatigue and burnout [15,16]. Therefore, further research is needed to evaluate how providers respond to EHR notifications, whether notifications lead to desired outcomes, specifically ACP conversation and trial recruitment in this study, and provider likelihood of continuing to use these notifications. The aim of this study is to evaluate an embedded EHR notification to identify patients appropriate for ACP in primary care clinics at 1 health system participating in the Meta-network Learning and Research Center (Meta-LARC) ACP study; we report provider usage and perspectives on this notification tool (hereafter referred to as the Meta-LARC ACP Notification substudy).

## Methods

### Study Design

The Meta-LARC ACP study is a Patient-Centered Outcomes Research Institute (PCORI)–funded US-Canadian multisite cluster randomized trial comparing clinician-focused to team-based implementation of the Serious Illness Care Program (SICP) developed by Ariadne Labs. The clinician-based arm focused on the primary care clinician leading to ACP conversations, and the team-based arm used the primary care team (including nurses, medical assistants, and clinicians) to have ACP conversations [17]. Each site determined which specific methods to implement to identify patients appropriate for ACP.

Within Duke Health, an EHR notification was developed to identify patients who would be appropriate for ACP, remind clinicians to have ACP, as well as to refer patients to the Meta-LARC ACP study. Duke Health is an academic health system serving urban, suburban, and rural populations in the Piedmont region of North Carolina. In the Duke Primary Care clinics, patients are assigned to a usual provider. If the provider is unavailable (eg, urgent same-day visit), then a patient may see any available provider.

Six Duke Primary Care clinics participated—3 clinics in the clinician-based arm and 3 clinics in the team-based arm—from January 1, 2019, to January 31, 2021. Study recruitment was initially set to end in June 2020 but was extended through January 2021 due to the COVID-19 pandemic. A total of 45 clinicians were trained with Serious Illness Care Program, participated in the study, and received EHR notifications. This included 32 physicians (Doctor of Medicine or Doctor of Osteopathic Medicine), 11 advanced practice practitioners, and 2 population health nurses.

The objectives of the Meta-LARC ACP Notification substudy are to (1) describe the implementation and usage of the EHR ACP notifications, (2) assess provider interactions with the EHR notifications, (3) evaluate the association of notifications with study referrals and with occurrence and documentation of ACP conversations, and (4) elicit provider perspectives on the EHR notification.

### Development of ACP Notification Tool in the EHR

**Figure 1:**
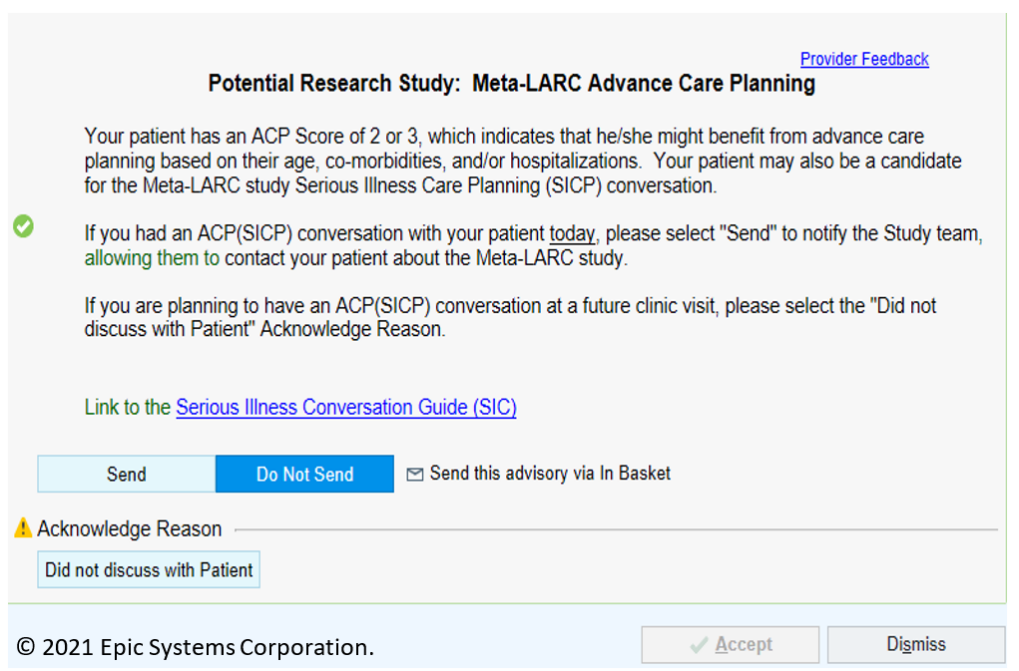
Advance care planning (ACP) notification. Meta-LARC: Meta-network Learning and Research Center.

We used an ACP scoring system developed at Duke Health to identify patients who may benefit from an ACP conversation. The ACP score adds 1 point for each of the following: (1) age at or older than 70 years, (2) three or more prespecified comorbidities using Systematized Nomenclature of Medicine (SNOMED) clinical terms, and (3) two or more hospitalizations within the health system in the past year [18,19]. Patients with an ACP score of 2 or 3 were considered more likely to benefit from an ACP conversation and were flagged for this study. This ACP score was embedded in the EHR, and a notification (a Best Practice Advisory within the Epic EHR system; Figure 1) appeared when the chart was opened during the encounter with identified patients. As prior diagnoses and hospitalizations were needed to calculate the ACP score, notifications would alert established patients meeting the ACP score criteria. Also, the EHR algorithm did not exclude patients with prior ACP documentation.

**Figure 2:**
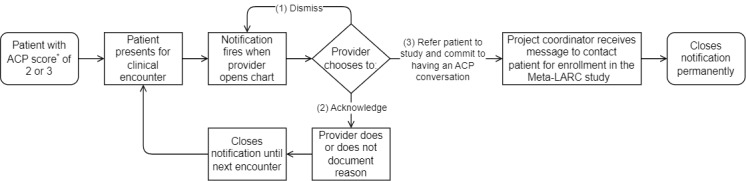
Process map with advance care planning (ACP) notification process and provider actions. *ACP score identified patients who may benefit from an ACP conversation. There are a total of 3 points, with 1 point for each of the following: age at or older than 70 years, three or more prespecified comorbidities using Systematized Nomenclature of Medicine clinical terms, and two or more hospitalizations within the health system in the past year. Meta-LARC: Meta-network Learning and Research Center.

From May 14, 2019, to January 14, 2021, the notification alerted enrolled providers when the patient presented for their clinic visit and the encounter visit was opened in the EHR. Once the notification appeared, providers were able to perform one of three actions: (1) dismiss the notification temporarily and have it retrigger when the chart was opened again in the same encounter, (2) acknowledge and remove the notification for the encounter, or (3) refer the patient to the study and commit to having an ACP conversation with the patient (Figure 2). If providers acknowledge the notification, providers also have an option of documenting a reason for the action. Provider responses to the notification (dismiss, acknowledge, or referral to study) were measured. While EHR notifications were the primary reminder for providers to have ACP conversations and occurred in the moment during the clinic visit, providers also received weekly emails with a list of their patients who had an ACP score of 2 or 3 and monthly practice facilitation visits during the enrollment period.

Notifications that occurred outside of the 6 primary care clinics were excluded from the analysis (n=511). These excluded notifications occurred among providers (N=8) who had transitioned out of the primary care clinic and resident trainee providers (N=9) who also practiced outside of the primary care clinic (eg, inpatient medicine service).

### Data Collection and Outcomes

EHR data were extracted over the study period; additional 2 weeks of data after the conclusion of the notification were included to provide time for providers to act on the notification. These data included provider action to notification (dismiss, acknowledge, and refer patient to study) and any free-text notification comments; patient demographics (age at start of study period, sex, race, and ethnicity); health care utilization (primary care visits and hospitalizations during notification period); primary insurance payor, ambulatory referrals to hospice, and death; and ACP documentation. ACP documentation included ACP notes; legal documents uploaded to the EHR, such as advance directives, health care power of attorney forms, or Physician Orders for Life-Sustaining Treatment (POLST) forms; or the use of ACP billing codes (Current Procedural Terminology [CPT] 99497 and 99498). No specific template was recommended for ACP note documentation; health system templates were available and Epic SmartPhrase codes were used to track documentation in the medical record [20]. POLST is a portable medical order form addressing specific health care treatments in more detail compared to advance directives and Do-Not-Resuscitate orders. ACP notification tool metrics regarding provider usage and response were also extracted from the EHR. For insurance payors, we grouped Medicare and Medicare Advantage into 1 Medicare group. Medicaid pending, in-state Medicaid, and out-of-state Medicaid were in 1 group. All other payors were grouped as commercial and other. Primary care visits were counted as any visit to a primary care clinic.

Our primary outcome was referral to the study through the EHR notification. Our secondary outcomes were documentation of ACP in the EHR, as indicated by the presence of ACP notes, and usage of related billing codes.

### Notification Free-Text Comments

We also evaluated an additional free-text response outcome for providers not referring patients. When providers chose to acknowledge responses, providers had an opportunity to leave free-text comments in the notification. Free-text comments were not required to complete the notification. An inductive approach was used to summarize major themes in free-text comments, which included lack of time, not claiming responsibility for the conversation, high visit acuity, and inappropriate timing.

### Provider Survey

In August 2020 and during the COVID-19 pandemic, approximately 1 year after the implementation of the ACP notification, we distributed a self-administered, closed and open-ended questionnaire soliciting provider perspectives regarding the ACP notification. We asked participants to rate their satisfaction (strongly disagree, disagree, agree, or strongly agree) using the ACP notification, its impact on the delivery of care, and its accuracy in identifying patients for ACP, as well as whether the provider would recommend the implementation of this notification for others. Meta-LARC providers were invited by email to a web-based survey using Qualtrics. A reminder email was sent 2 weeks later. The survey was open for 6 weeks.

### Statistical Analysis

Descriptive statistics were used for patient and provider characteristics, provider reaction to the ACP notification, and survey results. Results were presented in the following groups: (1) patients referred to the Meta-LARC ACP study, (2) patients who were not referred to the Meta-LARC ACP study, and (3) all patients identified by the ACP notification. Two patients were referred more than once, and each was counted as 1 referral per patient. Percentages were calculated for dichotomous and categorical data and mean and SD or median and IQR for continuous variables. We describe the frequency of occurrence of documentation related to ACP, advance directives, health care power of attorney, or POLST forms. Health care utilization (primary care visits and hospitalizations) were also described.

A logistic regression was performed using deciles to identify when a significant change in number of notification and referral to study occurred. As the deciles had overlapping notification values, we categorized the notifications into 9 categories (1, 2, 3, 4, 5, 6, 7 to 8, 9 to 13, or greater than 13 notifications). It was found that at 6 notifications there was a significant change in referral response (Multimedia Appendix 1). Therefore, we dichotomized notifications as less than or equal to 5 and greater than 5.

The chi-square test was used to compare associations between notifications (5 or less notifications and greater than 5 notifications) and (1) referral to the study, (2) ACP note documentation, and (3) ACP billing. We also examined associations between referral to study and (1) ACP documentation and (2) ACP billing. Associations where the primary care provider had an active role in (eg, referral response in the notification, note documentation, and billing) were only evaluated. Therefore, we did not compare associations with advance directives, health care power of attorney, or POLST forms, as these can be placed or submitted by patients and providers outside of the primary care clinic. Lastly, baseline patient characteristics and association with referral to study were evaluated using simple logistic regression models and complete data. Reference groups for the analysis were age (<65 years old), race (White), and sex (female). We excluded ethnicity, as the majority of patients were non-Hispanic, and insurance status, as the majority of patients were Medicare. Odds ratio (OR), CI around OR, and *P* values were reported. Analyses were performed using R (version 4.0.3; R Foundation for Statistical Computing) [21].

### Ethics Approval

The Meta-LARC ACP study was reviewed and approved by the Trial Innovation Network Central Institutional Review Board (IRB) at Vanderbilt University (VUMC IRB#181084) for the sites in the United States. As this study analyzes data without additional primary data collection, this secondary analysis was approved by the Duke University IRB (Pro00106329) with a waiver of consent. Data include protected health information data, which are stored in secure university-approved systems. There was no compensation for this additional analysis.

## Results

### Overview

Over the course of study period, 2877 unique patients were identified by the ACP score and triggered an ACP notification, and 45 providers received a total of 17,047 notifications. Patients identified by the ACP score were on average 78.0 (SD 10.5) years old, and the majority were female (n=1678, 58.3%), White (n=1868, 64.9%), and non-Hispanic (n=2822, 98.1%), with Medicare as the primary insurer (n=2629, 91.4%). A total of 290 unique patients (10.1%) were referred to the study by the notification. As of May 25, 2022, a total of 733 (25.5%) of patients identified have died (Table 1).

**Table 1:** Characteristics of patients eligible for advance care planning (ACP) and those referred to the study.

			No referral to study (N=2587, 89.9%)	Referral to study (N=290, 10.1%)	Overall (N=2877)
**Patient characteristics**					
	Age, mean (SD)		76.9 (10.7)	78.1 (9.2)	77.1 (10.6)
	**Gender, n (%)**				
		Female	1511 (58.4)	167 (57.6)	1678 (58.3)
		Male	1076 (41.6)	123 (42.4)	1199 (41.7)
	**Race, n (%)**				
		White	1694 (65.5)	174 (60.0)	1868 (64.9)
		Black	813 (31.4)	109 (37.6)	922 (32.0)
		Mixed race or other	66 (2.6)	6 (2.1)	72 (2.5)
		Missing	14 (0.5)	1 (0.3)	15 (0.5)
	**Ethnicity, n (%)**				
		Non-Hispanic	2537 (98.1)	285 (98.3)	2822 (98.1)
		Hispanic	11 (0.4)	0 (0)	11 (0.4)
		Missing	39 (1.5)	5 (1.7)	44 (1.5)
	**Primary insurance payor, n (%)**				
		Medicare	2364 (91.4)	265 (91.4)	2629 (91.4)
		Medicaid	31 (1.2)	6 (2.1)	37 (1.3)
		Other	167 (6.5)	17 (5.9)	184 (6.4)
		Missing	25 (1.0)	2 (0.7)	27 (0.9)
**Notifications**					
	Notifications per patient, median (IQR)		4 (2-8)	2 (1-5)	4 (2-8)
	**Provider response to notifications per patient, median (IQR)**				
		Acknowledge	2 (1-4)	0 (0-2)	1 (1-4)
		Dismiss	1 (0-5)	0.5 (0-2)	1 (0-4)
**Health care** **utilization**					
	**Primary care provider visits, median (IQR)**		5 (3-8)	7 (4-9)	5 (3-8)
		Missing, n (%)	28 (1.1)	1 (0.3)	29 (1.0%)
	Hospitalizations (≥2 during study period), n (%)		604 (23.4%)	60 (20.7)	665 (23.1)
	Hospice referral, n (%)		223 (8.6%)	39 (13.4)	262 (9.1)
	Death, n (%)		643 (24.9%)	90 (31.0)	733 (25.5)
**ACP documentation**					
	ACP note in EHR^a^, n (%)		188 (7.3)	125 (43.1)	313 (10.9)
	**ACP billing, n (%)**				
		99497	4 (0.2)	23 (7.9)	27 (0.9)
		99498	1 (0.0)	1 (0.3)	2 (0.1)
	Health care power of attorney, n (%)		123 (4.8)	20 (6.9)	143 (5.0)
	Advance directives, n (%)		56 (2.2)	18 (6.2)	74 (2.6)
	POLST^b^, n (%)		34 (1.3)	5 (1.7)	39 (1.4)

^a^EHR: electronic health record.

^b^POLST: Physician Orders for Life-Sustaining Treatment.

### Provider Actions and Responses

Of the 17,047 notifications, the most common response to the notification was dismiss (n=9496, 55.7%), followed by acknowledge (n=7251, 42.5%), and referral to study (n=292, 1.7%). Of note, referral to study should only occur after the provider initiated an ACP conversation, therefore, providers can dismiss or acknowledge the notification until the ACP conversation occurs at a future visit. There was a median of 4 (IQR 2-8) notifications per patient. The maximum number of notifications for any individual patient was 78.

In total, providers received a median of 269 (IQR 65-552) notifications over the study period. Of those, providers dismissed a median of 62 (IQR 16-179) times, acknowledged a median of 40 (IQR 11-191) times, and referred to the study a median of 2 (IQR 0-10) times. Providers had a median of 61 (IQR 27-135) patients identified by the study algorithm.

Free-text comments on acknowledge responses were provided for 658 notifications with comments grouped into categories shown in Table 2. The 28 providers (28/45, 62%) who provided comments wrote a median of 3 (IQR 2-20) comments, where 1 provider wrote 332 comments. The most common reason provided was having insufficient time during the clinic visit, accounting for 337 of the 658 comments (51.2%). The other response categories each individually accounted for 8% or less of total responses, with the second most common reason being the provider did not think they were responsible for discussing ACP (53 of 658 comments, 8.1%). A small number of comments—39 of 658 (5.9%)—were associated with ACP being inappropriate for the identified patient.

**Table 2:** Provider comments for acknowledge responses (N=658).

Categories	Values, n (%)^a^
Lack of time	337 (51.2)
Provider was not responsible for conversation (eg, not the primary care provider or followed by oncology)	53 (8.1)
Acute visit (and commonly not primary care provider for the patient)	50 (7.6)
Inappropriate timing (eg, new patient, posthospital, or want to plan for a future visit)	49 (7.4)
Advance care planning previously discussed^b^	40 (6.1)
Inappropriate for advance care planning	39 (5.9)
Patient was not seen for a routine medical visit (eg, lab visit or telephone encounter)	30 (4.6)
Surrogate decision maker not present (eg, dementia)	26 (4.0)
Patient not interested	25 (3.8)
Other (eg, technical issues)	9 (1.4)

**^a^**Percentage calculated from total written comments (N=658).

^b^Advance care planning reported on file for 8 patients.

A total of 290 patients (290/2877, 10.1%) were referred to the study by the notification. Among the 30 (30/45, 66%) providers who referred patients, these providers referred a median of 4.5 (IQR 2-15.75) patients each. The maximum number of patients referred by 1 provider was 45. Patients with greater than 5 notifications were less likely to be referred to the study (57/1092, 5.2% vs 233/1785, 13.1% of patients with 5 or less notifications; *P*<.001). Among patients with a notification and complete data (N=2802), Black patients had increased odds of referral to study (OR 1.34, 95% CI 1.04-1.73; *P*=.02). There was no significant difference in age or sex with referral to the study (Multimedia Appendix 2).

### ACP Documentation and Billing

Providers wrote 471 ACP notes for a total of 313 patients. A total of 34 providers (34/45, 75%) wrote an ACP note in the EHR with a median of 7.5 (IQR 3-15.5) notes per provider. The maximum number of notes written by 1 provider was 68 during the study period. Thirty-two patients had more than 1 ACP note. Patients were more likely to have an ACP note documented by a study provider if they were referred to the study (125/290, 43.0% referred vs 188/2587, 7.3% not referred; *P*<.001). There was no significant difference between number of notifications and ACP documentation (189/1785, 10.6% with 5 or less notifications vs 124/1092, 11.3% with greater than 5 notifications; *P*=.52).

Seven providers (7/45, 15%) used ACP billing codes (99497 or 99498) for a total of 24 times during the study period. Only 1 provider used the 99498 billing code during the study. Patients had increased likelihood of having ACP billing by a study provider if the patient was referred to the study (23/290, 7.9% referred vs 4/2587, 0.2% not referred; *P*<.001). There was no significant difference among patients who had greater than 5 notifications and ACP billing (19/1785, 1.1% with 5 or less notifications vs 8/1092, 0.7% with greater than 5 notifications, *P*=.43).

### Provider Survey Response

Eleven providers (11/45, 24%) responded to the survey. Results are presented as agree (agree or strongly agree) or disagree (disagree or strongly disagree). The majority of providers agreed that the notification improved delivery of care (7/11, 64%) was valuable to clinical care (7/11, 64%) and also accurately identified patients for ACP (8/11, 73%). While providers would recommend the notification for other clinics (7/11, 64%), fewer providers would continue to use the notification after the conclusion of the study (5/10, 50%). Despite the positive feedback, the notification was burdensome to clinical workflow (9/11, 82%) and difficult to navigate (6/11, 55%; Table 3).

**Table 3:** Survey results of 11 providers in the Meta-LAR^a^ ACP^b^ substudy.

	Strongly disagree	Disagree	Agree	Strongly agree
Improved delivery of care, n (%)	1 (9)	3 (27)	6 (55)	1 (9)
Valuable to clinical care, n (%)	1 (9)	3 (27)	6 (55)	1 (9)
Accuracy for identifying patients, n (%)	1 (9)	2(18)	3 (27)	5 (45)
Burdensome to clinical workflow, n (%)	0 (0)	2 (18)	7 (64)	2 (18)
Difficult to navigate, n (%)	2 (18)	3 (27)	5 (54)	1 (9)
Continue use after study concludes, n (%)	2 (20)	3 (30)	5 (50)	0 (0)
Recommend for other clinics, n (%)	1 (9)	3 (27)	7 (64)	0 (0)

^a^Meta-LARC: Meta-network Learning and Research Center.

^b^ACP: advance care planning.

### Participant Enrollment in Meta-LARC Study

The notification was implemented 5 months after the start of the overall Meta-LARC study; patients may have been referred outside of the notification prior to the implementation as well as during the study. Of the 113 patients who consented to the Meta-LARC study from this site, 100 patients (88.5%) were referred through the EHR notification. Six patients, while identified by the ACP score, were referred outside of the EHR notification. Seven patients were not identified by the ACP score and were referred to the study team outside of the EHR notification.

## Discussion

### Principal Findings

This Meta-LARC ACP Notification substudy implemented an embedded EHR notification to identify patients appropriate for ACP, referral to a clinical trial, and encourage ACP conversations and documentation. Approximately 10% of the patients identified by the EHR notification were referred to the study. In addition, we found that by the time providers received more than 5 notifications, providers were less likely to refer patients to the study. There was no significant difference in association among patients with more notifications (greater than 5 notifications) and ACP documentation or billing. While providers found the notification added value to their clinical care, the notification was difficult to use and was burdensome to clinical workflow.

### Comparison With Prior Work

Clinical trials can depend on provider participation for trial recruitment [22], and within the Meta-LARC study, providers would evaluate and refer patients to the trial. In this substudy, we successfully implemented a health system–specific ACP score to identify patients and an EHR notification to alert providers to potential eligibility and facilitate referral to the trial. The majority of patients were referred after notification implementation, which occurred in the fifth month of the year-long trial. This is similar to work done by Embi et al [13] demonstrating increased study referral after implementing automated notifications.

In addition to being a tool for increasing clinical trial referrals, the EHR notification encouraged providers to engage patients in ACP. The use of an embedded provider EHR notification has been demonstrated in other trials related to ACP in primary care [11,23]. Conversations on ACP are difficult to measure as providers may not document all conversations. Similar to other studies, we use ACP documentation as a surrogate to measure ACP conversations [24,25]. In this substudy, ACP documentation did not change with the number of notifications; in comparison, ACP was more likely documented among patients whom providers referred to the study.

The number of notifications did not influence the likelihood of having an ACP note documented because of other barriers to conducting ACP in a clinic setting. A study by Tung et al [23] used a clinical decision support system to identify patients for ACP and notify providers. In that study, all providers and patients received a recommendation for ACP. Patients at 2 clinics who received a resource packet prior to the visit were 5 times more likely to complete an advance directive compared to patients who did not receive a packet (21% vs 4%) [23]. While an embedded ACP EHR notification can overcome barriers in clinical practice by identifying appropriate patients, additional interventions for patients, such as patient-facing notifications and resource packets or a nurse navigator, may be needed to increase patient-provider ACP conversations [23,25,26].

Provider experiences of the ACP notification were predominantly positive. Few comments suggested inappropriate patient identification. Despite the positive response, providers found that the notification occurred too often, was burdensome to the daily work, and was difficult to navigate. This is likely related to the increased number of clicks required to process the notification in the midst of trying to care for a patient during a busy clinical day. If the provider “dismissed” the notification, the notification would reappear during the same encounter. These recurrent notifications can contribute to alert fatigue and increase provider workload and notification desensitization [27-31].

Furthermore, there are diminishing returns for providers to refer a patient to the study or conduct ACP after 5 notifications. Patients referred to the study were more likely to have less notifications because the notification would stop once the patient was referred to the study. The higher number of notifications for patients not referred to the study appears to be a marker of providers not acting upon or disregarding the notification. More concerning is that the additional notifications are less likely to achieve the original aim of the provider user. When additional notifications no longer prompt the provider action, these notifications may be setting the stage for alert fatigue, where repeated reminders may not increase ACP conversations and instead contribute to provider notification burden. Therefore, implementation of an EHR notification for ACP must be designed with the end user in mind, particularly in the context of the additional demand placed on provider’s attention and to reduce overall burden [32-34]. One potential improvement to this ACP notification may include a limit on the number of notifications over a set period of time. Since providers are less likely to respond after 5 notifications, the notifications might ideally turn off at that time with potential to retrigger in the future—perhaps after 1 year.

### Limitations

This substudy has a number of limitations. Less than a quarter of providers completed the electronic survey, and 1 provider provided half of the responses within the ACP notification. While response bias may limit conclusions drawn from provider perspectives on the notification, anecdotal provider feedback (from ongoing quarterly site visits conducted as part of the larger Meta-LARC study) has been similar to the feedback observed in the survey and comments. In addition, generalizability of the ACP score and notification may be limited. The EHR notification used an internally developed algorithm to identify primary care patients appropriate for ACP and is specific to our health system’s EHR (Epic) and its current user interface. Additional research is needed to validate the use of the ACP score among the primary care patient population, and this could also increase the generalizability of this algorithm to other primary care clinics within and potentially outside the health system. Lastly, the study design was limited to assessing the provider perspective on the ACP notification and did not collect data on the patient experience of care with the study providers. Future work as part of the Meta-LARC study will be evaluating the patient experience with ACP.

### Conclusions

The findings of this substudy support the implementation of using an EHR notification to both identify patients for clinical intervention—in this case, ACP—as well as for referral to a clinical study. This substudy highlights how the timing and repetition of the notification influence provider reception and actions. Additional research is needed to better understand how EHR notifications can be optimized for the provider user and improve overall implementation and adoption of EHR notifications for study referrals and ACP.

## Appendix

Multimedia Appendix 1 Logistic regression for number of notifications per patient and association with referral to study (N=2877).

Multimedia Appendix 2 Unadjusted logistic regression among patients with electronic health record notification and without missing data and association of patient characteristics for referral to study (N=2802).

## Abbreviations

ACP: advance care planning
CPT: Current Procedural Terminology
EHR: electronic health record
IRB: Institutional Review Board
Meta-LARC: Meta-network Learning and Research Center
OR: odds ratio
PCORI: Patient-Centered Outcomes Research Institute
POLST: Physician Orders for Life-Sustaining Treatment
SICP: Serious Illness Care Program
SNOMED: Systematized Nomenclature of Medicine

## Data Availability

The data sets generated and analyzed during this study are not publicly available due to privacy restrictions but are available from the corresponding author upon reasonable request.
