# Supplementary material for: Provider Interaction With an Electronic Health Record Notification to Identify Eligible Patients for a Cluster Randomized Trial of Advance Care Planning in Primary Care: Secondary Analysis
Source: J Med Internet Res. 2023 May 12;25:e41884. doi: 10.2196/41884 (PMC10221493; doi:10.2196/41884)
Supplement: Multimedia Appendix 1 [file jmir_v25i1e41884_app1.docx]

**Multimedia Appendix 1.** Logistic regression for number of notifications per patient and association with referral to study (N=2877).

| **Number of Notifications per Patient** | **Number of Patients** | **Odds Ratio for Referral** | **95% Confidence Interval** | ***P* value** |
| --- | --- | --- | --- | --- |
| 1 | 575 | Reference | - | - |
| 2 | 410 | 1.08 | 0.75, 1.53 | .68 |
| 3 | 321 | 0.70 | 0.45, 1.06 | .10 |
| 4 | 243 | 0.87 | 0.55, 1.34 | .53 |
| 5 | 236 | 0.61 | 0.36, 0.98 | .051 |
| 6 | 190 | 0.51 | 0.28, 0.88 | .021 |
| 7 to 8 | 303 | 0.40 | 0.23, 0.65 | <.001 |
| 9 to 13 | 315 | 0.34 | 0.19, 0.57 | <.001 |
| over 13 | 284 | 0.13 | 0.05, 0.27 | <.001 |
