# Supplementary material for: Provider Interaction With an Electronic Health Record Notification to Identify Eligible Patients for a Cluster Randomized Trial of Advance Care Planning in Primary Care: Secondary Analysis
Source: J Med Internet Res. 2023 May 12;25:e41884. doi: 10.2196/41884 (PMC10221493; doi:10.2196/41884)
Supplement: Multimedia Appendix 2 [file jmir_v25i1e41884_app2.docx]

**Multimedia Appendix 2.** Unadjusted logistic regression among all patients with electronic health record notification association of patient characteristics for referral to study (N=2802).

| **Characteristic** | **Odds Ratio for Referral** | **95% Confidence Interval** | ***P* value** |
| --- | --- | --- | --- |
| Age |  |  |  |
| < 60 years | — | — |  |
| 60-70 years | 0.98 | 0.46, 2.20 | > .90 |
| 70-80 years | 1.60 | 0.88, 3.19 | .20 |
| 80-90 years | 1.75 | 0.95, 3.53 | .09 |
| > 90 years | 1.33 | 0.62,2.96 | .50 |
| Sex |  |  |  |
| Female | — | — |  |
| Male | 0.99 | 0.77,1.27 | > .90 |
| Race |  |  |  |
| Caucasian | — | — |  |
| Black | 1.34 | 1.04,1.73 | .024 |
| Mixed/Other | 0.64 | 0.19, 1.57 | .40 |
